# Intention to Lose Weight, Weight Changes, and 18-y Mortality in Overweight Individuals without Co-Morbidities

**DOI:** 10.1371/journal.pmed.0020171

**Published:** 2005-06-28

**Authors:** Thorkild I.A Sørensen, Aila Rissanen, Maarit Korkeila, Jaakko Kaprio

**Affiliations:** 1Danish Epidemiology Science Centre, Institute of Preventive MedicineCopenhagen University Hospital, CopenhagenDenmark; **2**Obesity Research Unit, Helsinki University Central HospitalHelsinkiFinland; **3**Department of Public HealthUniversity of HelsinkiFinland; **4**Department of Mental Health, National Public Health InstituteHelsinkiFinland; Children's Hospital BostonUnited States of America

## Abstract

**Background:**

Weight loss in the obese improves risk factors for cardiovascular diseases and diabetes. However, several studies have shown inconsistent long-term effects of weight loss on mortality. We investigated the influence on mortality of intention to lose weight and subsequent weight changes among overweight individuals without known co-morbidities.

**Methods and Findings:**

In 1975, a cohort of individuals reported height, weight, and current attempts (defined as “intention”) to lose weight, and in 1981, they reported current weight. Mortality of the 2,957 participants with body mass index ≥ 25 kg/m^2^ in 1975 and without pre-existing or current diseases was followed from 1982 through 1999, and 268 participants died. The association of intention to lose weight in 1975 and actual weight change until 1981 with mortality was analysed while controlling for behavioural and psychosocial risk factors and hypertension as possible confounders. Compared with the group not intending to lose and able to maintain stable weight, the hazard ratios (with 95% confidence intervals) in the group intending to lose weight were 0.84 (0.49–1.48) for those with stable weight, 1.86 (1.22–2.87) for those losing weight, and 0.93 (0.55–1.56) for those gaining weight. In the group not intending to lose weight, hazard ratios were 1.17 (0.82–1.66) for those who did lose weight, and 1.57 (1.08–2.30) for those gaining weight.

**Conclusion:**

Deliberate weight loss in overweight individuals without known co-morbidities may be hazardous in the long term. The health effects of weight loss are complex, possibly composed of oppositely acting processes, and need more research.

## Abstract 2

Deliberate weight loss in overweight individuals without known illness is not obviously beneficial, and may be hazardous in the long term.

## Introduction

Weight loss in overweight and obese individuals leads to rapid improvement of the cardiovascular risk factor profile and reduced risk of developing type 2 diabetes. These observations have emerged from several studies of the general population [1–5] as well as from planned intervention studies [5–9]. In contrast, weight gain is associated with a worsened cardiovascular risk profile, increased risk of cardiovascular diseases and type 2 diabetes, and greater mortality from cardiovascular diseases and from all causes [5,10–13]. It would therefore be expected that overweight or obese individuals who lose weight would benefit from the effects leading to reduction or elimination of the excess mortality associated with overweight and obesity, which is largely attributable to cardiovascular disease and type 2 diabetes [5]. However, several prospective, long-term population-based studies have shown that weight loss, compared with stable weight in overweight or obese participants, is associated with future excess or unchanged mortality [10–13]. Moreover, cardiovascular diseases remain the main cause of mortality, even in studies that have taken other pertinent risk factors into account and eliminated confounding by diseases known to cause both weight loss and increased mortality [10–13].

The prevailing hypothesis to account for this apparent paradox is that the epidemiological studies have been unable to remove important confounding from several possible sources [14–18]. The excess mortality could be due to admixture of participants in the study populations with clinically manifest but undiagnosed diseases, sub-clinical diseases, or high-risk conditions or behaviours associated with both unintentional weight loss and excess mortality. Similarly, there may be participants with diseases or with high-risk conditions who have undertaken intentional weight loss in the hope of improving their health but who failed to eliminate the excess mortality, leading to so-called confounding by indication. A number of recent studies have included retrospective questions about whether an achieved weight loss was intentional or not [15,19–22]. The morbidity and mortality following intentional weight loss in these studies are equivocal and inconsistent, and they may also suffer from a variety of biases and confounding by indication [17,18]. One study from Israel [12], which included information on dieting for medical or slimming purposes prior to weight changes, found excess mortality associated with weight loss throughout the range of initial weight, although less excess mortality in the obese range. There was excess mortality in those who lost weight irrespective of whether it had been intended, but the study did not address this aspect specifically in the healthy overweight and obese groups. It is noteworthy that, contrary to its original aim, the large-scale, long-term clinical trial “Swedish obesity subjects study” comparing bariatric surgery to conventional treatment of obesity, in spite of considerable weight loss, improvement of risk factors, and reduction of diabetes incidence, has not yet found a reduced mortality [6].

In view of the rapidly rising prevalence of overweight and obesity almost everywhere, it is of paramount public health importance to know the long-term effects on mortality of the prevailing attempts to lose weight [5,17,18]. Randomised trials may be the best study design to address the effects of intended weight loss in selected groups such as the very obese, and among overweight or obese individuals with major co-morbidities or other high-risk conditions. However, such trials may not be feasible for addressing the problem of long-term effects in overweight and obese individuals who try to manage their body weight and who are still otherwise healthy and therefore have a much lower mortality in later life.

In the present population-based cohort study, we investigated whether intention to lose weight, as judged from actual attempts to lose weight at one point in time, and subsequent weight change among overweight or obese individuals were associated with subsequent long-term excess mortality. We selected individuals for whom there was no available evidence of diseases possibly linked to excess mortality or who were otherwise at increased risk of death, and we took into account lifestyle-related risk factors and any changes in them during the period of weight change.

## Methods

**Figure 1:**
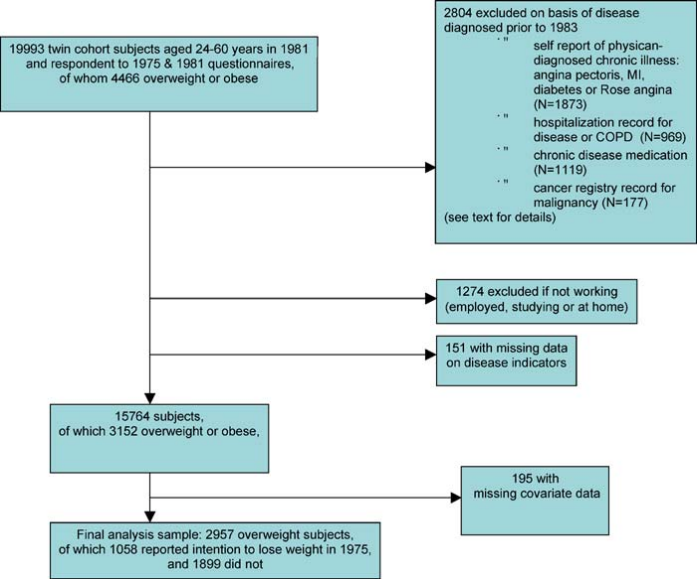
Flowchart Shows the Delineation of the Study Sample by Various Exclusions MI, myocardial infarction; COPD, chronic obstructive pulmonary disease

The study was based on The Finnish Twin Cohort, which was composed of all same-sex twin pairs born in Finland before 1958 in which both twins were alive in 1967 [23]. The participants received questionnaires in 1975 and in 1981 addressing height, weight, lifestyle issues, and physician-diagnosed diseases, and the 1975 questionnaire included additional items about current attempts to lose weight. The response rate in the 1975 survey was 89%. Among those still alive at the 1981 survey and who had responded in 1975, the response rate was 91%. The present study sample was derived from the 19,993 participants who were alive at the end of 1981, when ages ranged from 24 y through 60 y. The further delineation of the study sample is illustrated in Figure 1.

We aimed to exclude from the study sample participants suffering from any recognized chronic disease that could induce weight loss during 1975–1982 and subsequent increase in mortality. Thus, we excluded from the analyses participants who in the 1981 survey reported physician-diagnosed angina pectoris (*n* = 555), myocardial infarction (*n* = 170), or diabetes (*n* = 269), or who had angina pectoris according to standard chest pain history questions (*n* = 879). In addition, the cohort was linked to the national hospital discharge register, and participants with inpatient admissions for diabetes (*n* = 183), cardiovascular disease (*n* = 542) (except hypertension and venous disease), or chronic obstructive lung disease (*n* = 244) between 1972 and the end of 1982 were excluded. In addition, linking the cohort to a national drug prescription register allowed us to exclude participants who, based on medical certificates prior to 1983, had been granted reimbursable medication for somatic and psychiatric diseases. We excluded participants who had received prescriptions for all major chronic diseases (*n* = 1,119) except for hypertension. Presence of drug-treated arterial hypertension, as either reported in the 1981 survey or according to the medication reimbursement register, was rather common and therefore included as a covariate rather than used as an exclusion criterion. Participants who according to the Finnish Cancer Registry had diagnosed cancers before 1983 were excluded (*n* = 177). A total of 2,804 persons were excluded on the basis of known diseases. Finally, in order to take into account possible ill health not recorded otherwise, we excluded 1,274 participants who were not working in 1981 (“working” being defined as gainfully employed, working at home, or studying, and “not working” as being on early or disability pension (*n* = 356), unemployed (*n* = 461), or other (*n* = 457)), which is known to be associated with increased mortality.

Body mass index (BMI = weight/height^2^, kg/m^2^) was calculated from the reported height in the 1975 survey and from weight in both the surveys. Self-reported current height and weight generally have high accuracy [24,25], and the accuracy of the reports in the present study is supported by an analysis of another sub-study of The Finnish Twin Cohort [26]. The present analysis included only the participants who in 1975 were overweight or obese, defined as BMI ≥ 25 kg/m^2^, forming a total of 4,466 out of the 19,993 participants. These numbers were reduced to 3,152 out of 15,764 participants (with missing data in 151 participants) by the exclusions described above. Within this group, the median BMI was 26.7 kg/m^2^, the maximum BMI was 47.0 kg/m^2^, and 313 (9.9%) were obese (BMI ≥ 30 kg/m^2^). Recognizing that a change of 1.00 kg in body weight may have different implications for tall and short people, in analogy with body weight as such, we analysed weight changes as changes in BMI units rather than in kg. Weight loss and weight gain were analysed as separate continuous variables as well as categorical variables (the category weight loss was defined as any decline in BMI between 1975 and 1981, weight gain as an increase in BMI ≥ 1 kg/m^2^, and stable weight as no change in BMI or a gain less than 1 kg/m^2^).

In the 1975 survey, participants were asked whether they were currently trying to lose weight because of overweight, which was interpreted as “intention to lose weight,” and coded as yes/no. Then, if they were trying to lose weight, they were asked if they were doing so by voluntary restricting their food intake or changing their diet, increasing exercise, or by medication (coded yes/no in each case). No further details were requested, and these questions were not asked in the 1981 survey.

The lifestyle factors were recorded at both surveys and used in the analysis both as originally coded and recoded in simpler categories in order to avoid overloading the multivariate models. Smoking habits were originally coded as never smoker, occasional smoker (i.e., more than 10 packs of cigarettes in the lifetime, but never daily), former regular smoker, and current smoker. This variable was recoded as current smoker, yes/no. For alcohol drinking, the original question was used: five bottles of beer, a bottle of wine, or half a bottle of spirits on a single occasion at least once a month, yes/no; average alcohol consumption in g/d was also estimated. Physical activity was primarily assessed via an item on physical activity during leisure time with four response levels (i.e., equivalent to walking, alternating between walking and jogging, jogging, or running), but was recoded as engaging in physical activity more intense than walking, yes/no, which has previously proven adequate in relation to mortality analysis [27,28]. Life satisfaction was coded as being dissatisfied, yes/no, using a cut-point on a four-item dissatisfaction scale (range four to 20 points): yes if 12 or more, no if four to 11 [29]. Each of these risk factors were then categorized as being present both in 1975 and 1981, only in 1975, only in 1981, or absent at both surveys, which was coded by three dummy variables [28]. Work status (coded as gainfully employed, working at home, unemployed, studying, or other) and income (coded as eight levels) in 1975 were also included as potential confounders. Some of this information was missing in 195 participants, which led to the final sample size being reduced to 2,957 participants (Figure 1).

The participants were followed up until death, or the end of 1999, and 268 died. Underlying causes of death were available from Statistics Finland (http://www.stat.fi/index_en.html). The ascertainment of causes of death was based on forensic autopsy in 40% of cases. The age-sex adjusted mortality during follow-up of those included in the analysis was significantly lower than of all overweight participants replying to the two questionnaires (hazard ratio [HR] = 0.42, 95% confidence 0.36–0.49).

Twin analyses of the genetic and environmental contributions to BMI, change in body weight, intention to lose weight, and mortality have been carried out using the same cohort data [27,28,30,31]. The selection of the study sample for the present study implied that 2,067 participants out of the final sample of 2,957 did not have the co-twin included in the sample. Only six pairs were concordant for death, i.e., both died during the follow-up period. The within-pair correlations of phenotypes were adjusted for in the statistical analyses (see below).

### Statistical analysis

Total mortality from 1982 through 1999 was analysed by the Cox proportional hazards regression model with the number of days since the 1982 survey as the time scale using Stata software [32]. The estimated regression coefficients and standard errors were converted to HRs with 95% confidence intervals (CIs). Two types of models were analysed: a basic and a fully adjusted model. The basic model included sex and age (in 1981), an indication of whether the participants had attempted to lose weight (in 1975), the weight-change variables, and the BMI (in 1975). The validity of the model was assessed by running separate analyses, in which the covariates for sex, age, initial BMI (1975), and current smoking (1981) were each used for stratification; these analyses showed that the estimated effects of the variables of interest were reasonably consistent across the strata. In another series of analyses, we kept the covariates in the model together with the interaction terms between the variables of interest and the covariates, which showed that such interaction terms were not needed. The fully adjusted model additionally included the covariates for hypertension, smoking, alcohol drinking, physical activity, life satisfaction, work status, and income. For each of the covariates for which simplified recoding of the original variable was performed, models with the original and with the recoded version were estimated to assess if the simplified recoding showed indications of residual confounding of the effects of variables of interest, which was not the case. Therefore, only results from the analysis of the simplified, recoded covariates are presented. In both the basic and the fully adjusted models, age in 1981 was also used as a built-in stratification variable (age intervals 24–29, 30–34, 35–44, ≥45 y), allowing age to be modelled as a continuous variable within these age intervals as well.

In both types of models, we estimated HR for the *main effects* on mortality of intention to lose weight, and of weight change, and the separate effects of the 2 × 3 combinations of intention to lose weight and weight change, using the group not intending to lose weight and remaining stable in weight as the reference group (HR = 1.0). The intention to lose weight and the actual weight changes were then tested for *interaction* between them in their association to subsequent mortality (tested by the difference in global likelihood ratios of the models with and without the interaction terms). Thereafter, we estimated the differences in effects of weight changes within each of the groups intending and not intending to lose weight, and the differences in effects of intention versus no intention within each of the weight-change groups. The influences of the methods used to the weight-loss attempts were assessed by estimating the effects of weight loss versus stable weight among those who stated that they used diet as the method and among those using exercise as the method.

In order to evaluate the effects of sex, age, and follow-up time on the HR of interest, separate fully adjusted models were analysed within the two sexes, age strata < 45 and ≥ 45 y, the combination of sex and these age strata, and for the first 5 y of follow-up and the period thereafter.

The proportionality of hazards and linearity of effect of the continuous variables were checked. Using the available “cluster” option in the Stata program, correlated observations in twin pairs included in the final sample were adjusted for in the estimation of the standard errors of the HR and the *p* values. Two-tailed *p*-values below 0.05 were considered statistically significant. Using the Stata program, survival functions were estimated from the Cox regression models for the groups of interest while adjusting for relevant co-variables (sex, median age, and median BMI).

## Results

The numbers of participants were distributed by weight-loss intention in 1975 and weight change between 1975 and 1981 as shown in Table 1, which also shows their mean age at the start of mortality follow-up in 1981. Baseline BMI and the median and range of weight changes (in BMI units) in each group are shown in Table 2 for all participants and the 268 among the 2,957 participants who died during the follow-up. The weight changes were about the same irrespective of intention to lose weight and whether the participants died or survived the follow-up period.

**Table 1:**
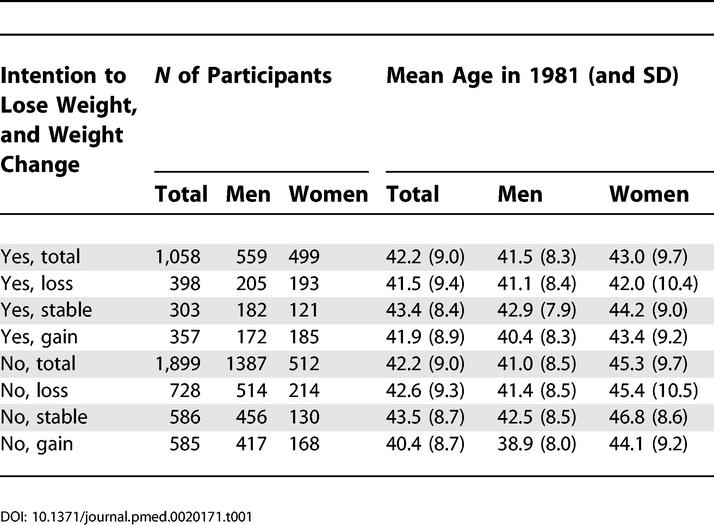
Number of Participants and Mean Age by Sex and Intention to Lose Weight in 1975 and Weight Change between 1975 and 1981 among 2,957 Overweight or Obese Participants (BMI greater than or equal to 25 kg/m^2^) Aged 24-60 y in 1981

**Table 2:**
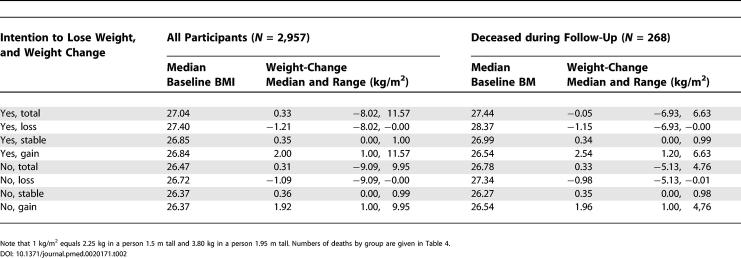
Median and Range of Baseline BMI by Intention to Lose Weight in 1975 and Weight Change between 1975 and 1981, and Median and Range of Weight Change (BMI units kg/m^2^) for Each Group among Overweight or Obese Participants (BMI greater than or equal to 25 kg/m^2^) Aged 24-60 y in 1981

Note that 1 kg/m^2^ equals 2.25 kg in a person 1.5 m tall and 3.80 kg in a person 1.95 m tall. Numbers of deaths by group are given in Table 4.

### Analysis of Main Effects of Intention to Lose Weight and of Weight Changes

Intention to lose weight as such had no influence on mortality in the follow-up period, the HR in the basic and fully adjusted models being 0.86 (CI 0.66–1.12) and 1.00 (CI 0.75–1.32), respectively, compared with no intention to lose weight (HR = 1.0).

Both those who gained weight and those who lost weight had increased mortality compared with the weight-stable group (HR = 1.0). In the basic and fully adjusted models, the respective HR for the weight losers was 1.43 (CI 1.06–1.92) and 1.40 (CI 1.04–1.90). For the weight gainers, the HRs were 1.46 (CI 1.06–2.02) and 1.38 (CI 1.00–1.92), respectively. Weight changes were also analysed as continuous variables (divided in weight loss and weight gain, the latter including the category defined as stable weight, i.e., with a gain of less than 1.00 kg/m^2^). The HR for weight loss per BMI units estimated in the two models was 1.12 (CI 1.00–1.26) and 1.11 (CI 0.99–1.24), respectively. For weight gain, the HRs were 1.13 (CI 1.02–1.25) and 1.11 (CI 1.00–1.23).

### Combined Analysis of Intention to Lose Weight and Actual Weight Changes

Table 3 combines intention to lose weight with the actual weight-change variable and compares each group with the group not intending to lose weight and able to maintain stable weight (HR = 1.0) during the 6 y from 1975 through 1981. There was significant excess mortality only in the group intending to lose weight in 1975 and who lost weight by 1981, and in the group not intending to lose weight and then gaining weight.

**Table 3:**
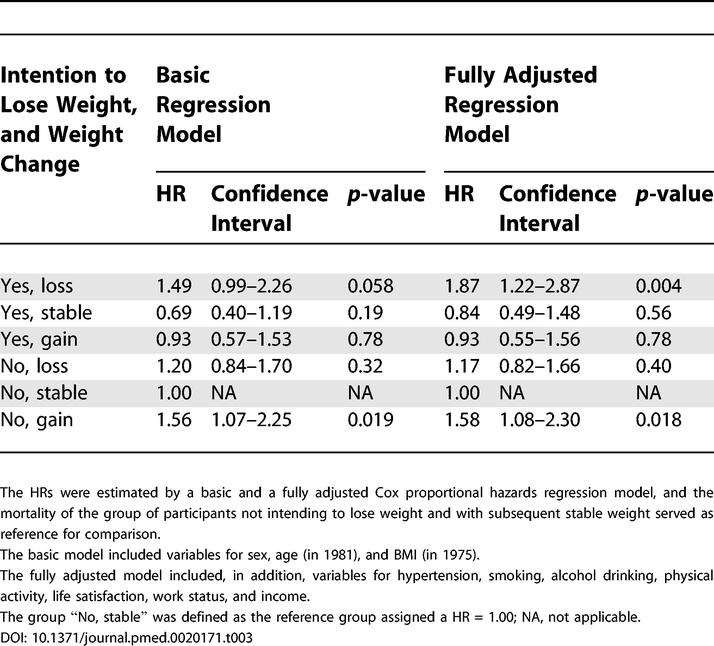
HRs with 95% Confidence Intervals of Total Mortality between 1982 and 1999 by Intention to Lose Weight in 1975 and Weight Change between 1975 and 1981

The HRs were estimated by a basic and a fully adjusted Cox proportional hazards regression model, and the mortality of the group of participants not intending to lose weight and with subsequent stable weight served as reference for comparison.

The basic model included variables for sex, age (in 1981), and BMI (in 1975).

The fully adjusted model included, in addition, variables for hypertension, smoking, alcohol drinking, physical activity, life satisfaction, work status, and income.

The group “No, stable” was defined as the reference group assigned a HR = 1.00; NA, not applicable.

The basic and fully adjusted models produced approximately the same estimates, except for the similar increases by adjustments in HR for the groups intending to lose weight and actually losing weight or maintaining it. The HR for mortality following intended weight loss increased from 1.49 to 1.87 and became statistically significant (*p* decreased from 0.06 to 0.004) with the adjustments of the fully adjusted model. In stepwise rebuilding of the model, it was found that adjustment for smoking increased the HR from 1.49 to 1.66, and further adjustment for physical activity increased the HR from 1.66 to 1.85, indicating the importance of these two variables as confounders; the group with intent to lose weight and actual weight loss smoked less and exercised more than other groups.

Except for this difference between the basic and the fully adjusted models, the two models produced essentially the same results, wherefore only the results from the fully adjusted models are presented in the following.

The lowest mortality was found in the group intending to lose weight but who maintained stable weight, the HR being 0.84, although this was not statistically significantly different from the reference group (HR = 1.0) of weight-stable participants who did not intend to lose weight (*p* = 0.56).

There was a statistically significant interaction between intention to lose weight (yes/no) and the weight-change variable (loss, stable, gain) (*p* = 0.014).

The distribution of causes of death (Table 4) did not differ significantly between the six groups (global chi-square test *p*-value = 0.89), and the deaths in the two groups exhibiting excess mortality showed no distinct differences compared with the distribution overall. The distribution of the causes of death was in accordance with that expected from concurrent national statistics for the same sex and age groups (data not shown).

**Table 4:**
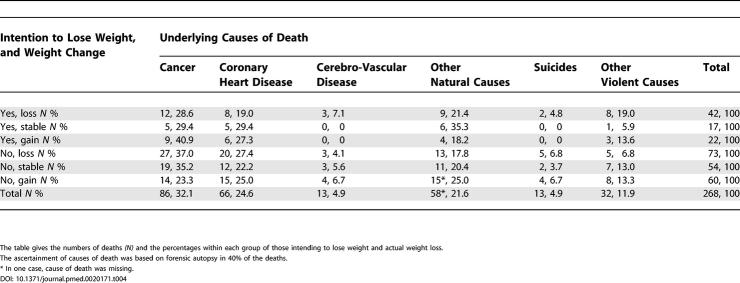
Distribution of Underlying Causes of Death among the 268 Overweight or Obese Participants Who Died during the Follow-Up Period 1982 through 1999 by Intention to Lose Weight in 1975 and Weight Change between 1975 and 1981

The table gives the numbers of deaths *(N)* and the percentages within each group of those intending to lose weight and actual weight loss.

The ascertainment of causes of death was based on forensic autopsy in 40% of the deaths.

* In one case, cause of death was missing.

### Analysis of Effects within Sex and Age Groups

The estimates of effects in the groups depicted in Table 3 showed generally the same pattern in men and women, in participants younger than 45 and participants 45 y or older, and in the three groups of younger and older men and older women (only 10 women younger than 45 y died, and none in the groups with no intention to lose weight and subsequent stable or increasing weight, so model-based estimates could not be produced). Table 5 shows that the estimated effects of weight change for those who intended to lose weight did exhibit the same pattern for the sex and age groups, and no statistically significant interactions by sex and/or age groups on mortality in the weight-loss group were found (all *p*-values > 0.23).

**Table 5:**
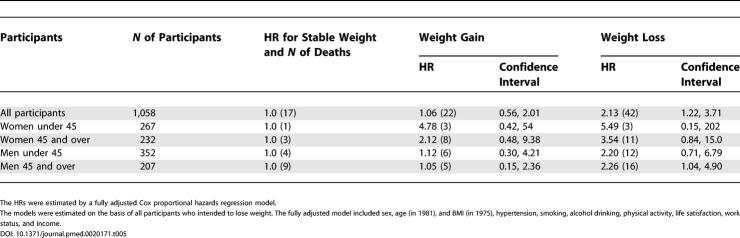
HRs with 95% Confidence Intervals of Total Mortality between 1982 and 1999 within the Group of Overweight or Obese Participants Intending to Lose Weight by Sex and Age Group, for Those Who Did Lose Weight or Gained Weight between 1975 and 1981 Compared with Those Who Maintained Stable Weight (Reference Group)

The HRs were estimated by a fully adjusted Cox proportional hazards regression model.

The models were estimated on the basis of all participants who intended to lose weight. The fully adjusted model included sex, age (in 1981), and BMI (in 1975), hypertension, smoking, alcohol drinking, physical activity, life satisfaction, work status, and income.

### Analysis of Effects of Weight Change within the Group Intending to Lose Weight

Among the participants intending to lose weight, those who lost weight showed excess mortality compared with those maintaining stable weight (HR = 1), the HR being 2.13 (CI 1.22–3.71). The corresponding HR for all cancer deaths as the outcome were 2.06 (CI 0.71–5.96), for all cardiovascular deaths, 2.04 (CI 0.64–6.49), and for all other natural causes, 1.31 (0.45–3.81). The participants who gained weight despite their intention to lose weight did not differ significantly from those who maintained weight, the HR being 1.06 (CI 0.56–2.01); also, none of the cause-specific outcomes were increased.

**Figure 2:**
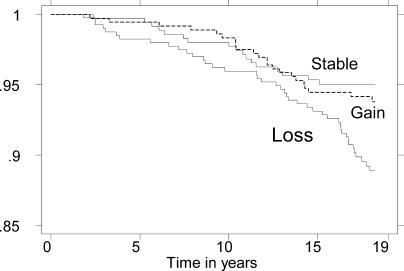
Mortality by Weight Change in 1975–1981 among Those Reporting Trying to Lose Weight in 1975 Probability of survival from baseline in 1982 through 1999 among 1,058 participants who in 1975 reported intention to lose weight and who either lost weight, gained more than 1.0 kg/m^2^ in BMI, or remained stable, i.e., were unchanged or gained less than 1.0 kg/m^2^ in BMI, between 1975 and 1981. The survival probability was adjusted using the Cox regression model for sex, median age, and median BMI. Note that the participants with weight loss had a lower survival rate throughout the 18 y of observation, whereas those with stable weight and weight gain did not differ.

As seen in Figure 2, the excess mortality of the intentional-weight-loss group was numerically greater during the first 5 y of follow-up, during which period the HR was 6.26 (CI 0.33–118). After the first 5 y of follow-up, a statistically significant excess risk was still observed, the HR being 1.88 (CI 1.05–3.39).

When analysing weight loss and any gain as continuous variables among those intending to lose weight, the HR per BMI units were 1.27 (CI 1.07–1.49) and 1.03 (CI 0.87–1.23), respectively.

### Analysis of Effects of Weight Change within the Group Not Intending to Lose Weight

**Figure 3:**
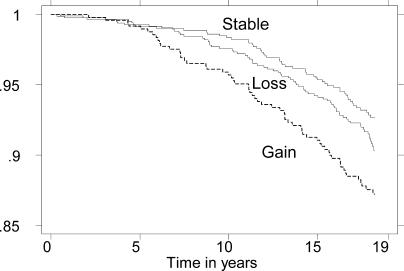
Mortality by Weight Change in 1975–1981 among Those with No Intention to Lose Weight in 1975 Probability of survival from baseline in 1982 through 1999 among 1,899 participants who in 1975 reported no intention to lose weight and who either lost weight, gained more than 1.0 kg/m^2^ in BMI, or remained stable, i.e., were unchanged or gained less than 1.0 kg/m^2^ in BMI, between 1975 and 1981. The survival probability was adjusted as in Figure 2. Note that the participants with weight loss had about the same survival rates throughout the 18 y of observation as those with stable weight, whereas those gaining weight showed a lower survival rate.

Among the participants not intending to lose weight, those who gained weight showed excess mortality compared with those with stable weight (HR = 1.0), with the HR being 1.64 (CI 1.12–2.42). Those who lost weight did not differ significantly from those with stable weight, the corresponding HR being 1.19 (CI 0.83–1.72). This pattern of mortality was apparent throughout the follow-up period (Figure 3).

When analysing weight loss and any gain as continuous variables among those not intending to lose weight, the HR per BMI units was 1.02 (CI 0.87–1.19) and 1.17 (CI 1.02–1.34), respectively.

### Analysis of Effects of Intention to Lose Weight within the Weight-Change Groups

Among the participants losing weight, those who intended to lose weight compared with those who did not intend to lose (HR = 1.0) showed a significantly increased mortality with a HR of 1.65 (CI 1.09–2.50) (in the basic model, the HR was 1.21 (CI 0.82–1.79), and this difference was due to the same confounding mentioned above and eliminated by the fully adjusted model).

Among participants who gained weight, those who had intended to lose weight had a lower mortality than those who did not intend to lose weight (HR = 1.0), the HR being 0.55 (CI 0.33–0.93). Participants who maintained stable weight had an insignificantly lower mortality if they had intended to lose weight, HR being 0.84 (0.49–1.48) (see Table 3).

### Analysis of Effects of Methods for Intended Weight Loss and Actual Weight Loss

The eventual weight losses observed in each of the groups employing the various methods for losing weight at the beginning of the observation period were quite similar in magnitude (Table 6). When dieting was the intended method, those who lost weight compared with those who maintained stable weight showed significant excess mortality (Table 6). When exercise was used as the method, the HR for those who lost weight compared with those who maintained weight was increased but not statistically significant.

**Table 6:**
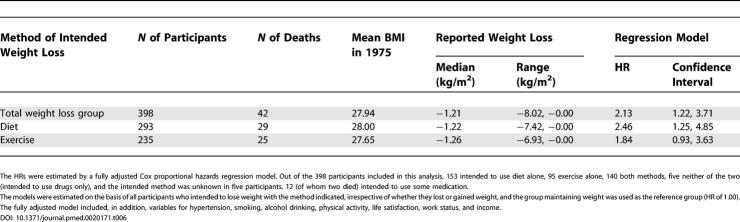
HRs with 95% Confidence Intervals of Total Mortality between 1982 and 1999 within the Group of Overweight or Obese Participants Intending to Lose Weight by Reported Methods of Weight Loss, for Those Who Did Lose Weight between 1975 and 1981 Compared with Those Who Maintained Stable Weight

The HRs were estimated by a fully adjusted Cox proportional hazards regression model. Out of the 398 participants included in this analysis, 153 intended to use diet alone, 95 exercise alone, 140 both methods, five neither of the two (intended to use drugs only), and the intended method was unknown in five participants. 12 (of whom two died) intended to use some medication.

The models were estimated on the basis of all participants who intended to lose weight with the method indicated, irrespective of whether they lost or gained weight, and the group maintaining weight was used as the reference group (HR of 1.00).

The fully adjusted model included, in addition, variables for hypertension, smoking, alcohol drinking, physical activity, life satisfaction, work status, and income.

## Discussion

In our study, both net weight loss and weight gain over a 6-y period among overweight or obese participants without known co-morbidities or high-risk conditions were associated with later increased long-term mortality, whereas attempts to lose weight at the beginning of this period in time, interpreted as intention to lose weight, by itself did not affect the long-term mortality. However, participants intending to lose weight and who experienced a net weight loss over the 6-y period had increased long-term mortality compared both with participants maintaining weight in spite of the intention to lose it and with participants with unintended weight loss. Those not intending to lose weight who later gained weight had increased mortality compared with participants maintaining stable weight and with participants gaining weight in spite of intending to lose weight.

Considering the arguments involved in the continuing controversy over the long-term health benefits of weight loss among overweight and obese individuals [14–18,22], the present study offers evidence of importance. Hitherto, excess long-term mortality after weight loss has been observed in several large-scale population-based prospective studies. Despite careful efforts to control confounding by risk factors and by underlying diseases associated with both weight loss and increased mortality, this excess long-term mortality has been attributed to inadequate control of such confounding [17,18]. The prevailing hypothesis has been that the obviously expected reduction in mortality after deliberate weight loss in the epidemiological observational studies has been masked by admixture of a large group of participants who, for various reasons, suffered from ill health and, as one sign thereof, had also lost weight.

A recent study, based on the National Health Interview Survey in the United States, addressed the question in a group of 6,391 overweight or obese participants at least 35 y old who were followed for 9 y after reporting on weight change and intention to lose weight during the past year [22]. Except for the finding that the lowest mortality was observed for those not trying to lose weight and who gained weight, the study yielded results in agreement with the prevailing expectation. It showed that intended weight loss compared with stable weight was associated with lower all-cause mortality, and that weight loss was associated with higher mortality only if it was unintentional. Furthermore, attempted weight loss as such was associated with lower mortality, independent of weight change; this suggests that confounding by medical indications was not a problem, but, on the other hand, that these participants may have been more healthy than those not intending to lose weight.

In this and most of the previous studies, the question about intention to lose weight was posed after the participants had achieved the weight loss, which implies a possible selection and recall bias. If overweight or obese participants who at the time of reporting are feeling healthy and have experienced a weight loss are more prone to consider that this was intentional, then a study design of this type may favour delineation of a particular group of participants with low morbidity and mortality [17].

Our study was based on a large sample of overweight and obese adult working-aged participants. We aimed to effectively minimize the effect of confounding due to underlying disease before and during the weight-loss period. Notably, those suffering from relevant illnesses, diagnosed by physicians and reported either by the participants themselves or in three national medical registers, were excluded or otherwise taken into account. The finding that those not intending to lose weight but who did lose weight did not experience excess mortality suggests that the exclusion of underlying diseases as a possible confounder was successful. In contrast to most previous studies on intentional weight loss [15,19–22], this study obtained the information about the intention to lose weight on the basis of a current attempt to lose weight before the actual subsequent weight changes were known and 6 y prior to the baseline of the follow-up. This timing eliminates the recall and selection bias that might hamper studies in which such information is obtained later. On the other hand, subsequent weight loss may not be a direct consequence of the intent to lose weight during the following 6 y, and similarly the weight loss among those not initially intending to lose weight may be due to a later intentional weight loss. These changes over time in possible causes of the weight loss imply that the observed associations in our study can be interpreted only as conservative predictions of the effects of intentional weight loss on long-term mortality. The finding that among those intending to lose weight mortality increased only if weight was actually lost, but not if it remained stable or increased after 6 y, suggests that the excess mortality following weight loss is not due to later regain in body weight.

The most likely behavioural and psychosocial risk factors that could have confounding effects—smoking habits, alcohol drinking, physical activity, life satisfaction, work status, and income—were included in the analysis. It is reassuring that the results (with a few exceptions) were essentially independent of the adjustment for these confounders in both the original versions and the simplified recoded versions, which minimizes the likelihood of residual confounding because of insufficient precision in their assessment. The fact that the study sample consisted of adult twin individuals is unlikely to have affected the results; twinship itself was taken into account in the statistical analysis. Moreover, the genetic influence on total mortality and its relation to body weight is small [33,34], and adult twins have the same overall mortality as singletons [35].

Several other aspects of the results qualify their interpretation. The findings that weight loss and weight gain, compared with stable weight, were both associated with increased mortality indicates that the present study sample of overweight individuals in this respect was similar to several others without overt disease in which the same relationships have been found [10–14]. The finding that intention to lose weight as such had no detectable effect on mortality suggests that this intention is not an indicator of the participants being especially concerned about health. In particular, it does not seem to be a marker of an overall healthier or, conversely, more hazardous lifestyle resulting in mortality different from that in participants not reporting attempts to lose weight. The finding that the excess mortality in the weight-loss group with initial intention to lose weight was present also after the first 5 y of observation and throughout the entire subsequent follow-up period also supports that the weight loss in the group intending to lose weight did not reflect effects of incident diseases or other health problem inducing the weight loss.

However, we cannot exclude the possibility that this group of participants was heterogeneous, with some being very healthy and health-conscious, and others for whom intention to lose weight was an indicator of insidious or imminent health problems. In view of the difficulties in maintaining deliberate weight loss during a 6-y period, the observation that this was in fact achieved could in itself be an indicator of the presence of underlying disease processes in some of the overweight or obese participants that could have motivated the intention to lose weight, facilitated its apparent success and maintenance, and led to later increase in mortality. If this is the explanation of the finding, then it must be symptomatic chronic diseases, ongoing for more than 6 y and escaping clinical diagnosis during this period in spite of the universal and practically free access to medical care in Finland. Moreover, the effect of such diseases on total mortality would have to be strong enough to completely eliminate any beneficial effects of intentional weight loss in the entire group and to be contributing to increased mortality throughout the observation period. Although this explanation would bring our results in line with the prevailing expectations, it seems implausible and possibly hazardous to rely upon.

The analysis of the causes of death did not indicate any single disease as responsible for all the excess mortality. Cause-of-death information has its well-known limitations, and we had relatively modest numbers by cause of death. It is likely that the effects of intentional weight loss is carried through to increased mortality by a more general deleterious effect on ability to cope with or resist the risks of incident diseases, which justifies that we focus on total mortality rather than cause-specific mortality.

One condition possibly contributing to the excess total mortality could be type 2 diabetes, which can remain undiagnosed for several years and may induce weight loss. However, analysis of supplementary information on later-onset diabetes suggests that occurrence of diabetes cannot explain the excess mortality among those intending to lose weight and who did lose weight (unpublished results, based on both a follow-up questionnaire in 1990, i.e., 8 y after the start of the follow-up, and on medical certificates for diabetes medication in the national drug prescription register for diagnoses covering 1983–1994). Another possibility is non-psychotic depression, but this was not supported by the analysis of causes of death, which showed no particular excess of suicides or of cardiovascular deaths. Moreover, the inclusion of the life satisfaction score, highly correlated to depression, in the analysis did not change the results.

Although we have no definite explanation of our finding of an excess mortality following intentional weight loss, we may consider some possibilities based on comparison with other studies [17]. Weight loss may release toxic substances from the fat tissue [36], but if this mechanism were important, we would expect excess mortality in the unintentional weight-loss group as well. We may assume that intentional weight loss as induced by deliberate calorie restriction has multiple biological effects—some potentially beneficial, some potentially harmful. The net effects on the health of participants may depend on their current condition. Thus, overweight and obese participants with clinically manifest or sub-clinical diseases such as type 2 diabetes may benefit from intentional weight loss, also in terms of mortality [37]. However, we may speculate that in overweight or obese participants without such co-morbidities or high-risk conditions, the harmful effects of intentional weight loss may predominate and lead to excess mortality. If so, the outcome of a study of the association of weight loss with mortality would then depend on the particular admixture of healthy and diseased overweight or obese participants. The privileged conditions for conducting this type of research in Finland may have secured the identification of a study sample with minimal co-morbidity, resulting in a more clear manifestation of the harmful effects of intentional weight loss and the lack of such effects of unintentional weight loss.

Intentional weight loss may induce loss of fat-free mass, which seems an unavoidable accompanying consequence [38–40]. In cohort studies with a single baseline measurement of fat mass and fat-free mass, mortality increases with the decrease in fat-free mass [41–44]. The interpretation is further supported by a detailed re-analysis of two large longitudinal population studies in the United States, addressing the relation between total mortality and weight change in comparison with the relation between total mortality and changes in skin-fold thickness as an indicator of changes in the amount of subcutaneous fat tissue [45]. This analysis showed that decline in skin-fold thickness (for a given weight loss) was associated with reduced mortality, whereas overall weight loss (for a given change in skin-folds) was associated with increased mortality.

Even if the method reported being used for the attempts to lose weight at the beginning of the observation period had not been sustained, the differences in mortality between them may provide useful suggestion for explanation of the overall findings. In the present study, the distribution of the excess mortality over many years of the follow-up period suggests that the reason is not the known hazardous effects of severely imbalanced dieting. Our finding that the excess mortality was worse in the group reporting using dieting than in the group reporting using exercise, is consistent with the possibly hazardous effects of loss of fat-free body mass. In large-scale population studies, the association of changes in lean body mass and physical activity is barely detectable [38]. It is likely that those reporting exercise as their weight-loss method had in fact been dieting to some extent, because it is very difficult to achieve weight loss by exercising alone [46,47]. On the other hand, using exercise as part of a controlled dieting-based weight-loss program may inhibit the tendency to lose fat-free body mass [39,48], which could explain why the deleterious effects were less in this group compared with those using diet. In this connection, it is important to note that in the present study sample, reported leisure exercise, as expected, had an overall strong beneficial effect on total mortality [27,28].

To understand the difference in mortality patterns associated with intentional and unintentional weight loss in the present study, we should also consider other mechanisms. On one hand, it seems plausible that unintentional weight loss may be associated with excess mortality in overweight or obese participants who suffer from some of the many diseases that may lead to both weight loss and increased mortality; these cases were excluded as effectively as possible. On the other hand, unintentional weight loss in overweight or obese participants without co-morbidities may be interpreted in a different way. We need to allow for the possibility that the size of the fat mass may change by mechanisms other than negative energy balance enforced by deliberate calorie restriction. Health-enhancing lifestyle changes such as increased physical activity or dietary modifications may lead to loss of body fat and increase in lean body mass. This may also be the explanation why weight gain in the group intending to lose weight was followed by a lower mortality than weight gain in the group not intending to lose weight. There appear to be still unknown factors contributing to weight fluctuations in healthy individuals, in whom weight changes in one direction were the strongest predictors of subsequent weight change in the opposite direction [49], i.e., prior weight loss best predicted future weight gain.

In conclusion, the long-term effects of weight loss are complex, and they may be composed of oppositely operating effects with net results reflecting the balance between these effects. We may cautiously suggest that future studies assess short-term advantages of planned weight loss against possible long-term risks, and assess if the optimal strategy among already overweight individuals could be to avoid further weight gain. If this is true, it puts a major emphasis on the need for prevention of development of overweight and obesity. This conclusion does not contradict the possible beneficial effects of planned weight loss in obese individuals who have already developed co-morbidities of their obesity, such as type 2 diabetes and symptoms of cardiovascular disease [50]. Obviously, more research will be needed to evaluate and explain our findings before they can be used as basis for advice about intentional weight loss in the large population of otherwise healthy overweight and obese individuals.

**Patient Summary**

**Background**

Although it seems obvious that when overweight people lose weight their health should improve, previous work has suggested that the relationship between weight loss and health may not be as simple as that. For example, it is difficult to control for all other possible things that might cause weight loss, such as other medical conditions that could then increase mortality.

**What Did the Researchers Do?**

They started with a population of 19,993 Finnish twins who were asked in 1975 about their weight and whether they intended to lose weight. In 1981, they were asked again about their weight, and then followed for up to 18 years to see if any died. The researchers took out of the analyses all the people who had illnesses, or those who had data missing. They analysed mortality against intention to lose weight in 1975 and actual change in weight. They found that those people who intended to lose weight and who actually did so had a slightly higher mortality than those who gained weight or whose weight remained the same. In people who did not intend to lose weight, gaining weight was associated with a slightly higher mortality.

**What Do These Results Mean?**

In the people studied here who were otherwise healthy and only moderately overweight, losing weight seemed to be associated with higher mortality. What makes these results quite difficult to interpret is that the actual number of people who died was not very high, but nonetheless intentional weight loss did not improve mortality. One reason for this result may be that when people diet to lose weight, they lose fat-free tissue as well as fat.

In people who have medical conditions related to obesity, losing weight is obviously desirable. But overall, preventing people, especially children, from becoming overweight in the first place seems crucial, since this work suggests that once weight is gained losing it again may not be good for health.

**Where Can I Get More Information?**

The National Institute of Diabetes and Digestive and Kidney Diseases (NIDDK) of the National Institutes of Health (NIH) has several pages of information on weight control: http://www.niddk.nih.gov/health/nutrit/nutrit.htm

The International Association for the Study of Obesity has a Web site with information on obesity worldwide: http://www.iotf.org/

## Abbreviations

BMI: body mass index
CI: confidence interval
HR: hazard ratio
